# Differential Expression of Toll-like Receptors in Dendritic Cells of Patients with Dengue during Early and Late Acute Phases of the Disease

**DOI:** 10.1371/journal.pntd.0002060

**Published:** 2013-02-28

**Authors:** Silvia Torres, Juan Carlos Hernández, Diana Giraldo, Margarita Arboleda, Mauricio Rojas, Jolanda M. Smit, Silvio Urcuqui-Inchima

**Affiliations:** 1 Grupo Inmunovirología, Sede de Investigación Universitaria, Universidad de Antioquia, Medellín, Colombia; 2 Department of Medical Microbiology, Molecular Virology Section, University Medical Center Groningen, University of Groningen, Groningen, The Netherlands; 3 Grupo Infettare, Facultad de Medicina, Universidad Cooperativa de Colombia, Medellín, Colombia; 4 Instituto Colombiano de Medicina Tropical, Universidad CES, Sabaneta, Antioquia, Colombia; 5 Grupo de Inmunología Celular e Inmunogenética, Instituto de Investigaciones Médicas, Facultad de Medicina, Universidad de Antioquia, Medellín, Colombia; University of Rhode Island, United States of America

## Abstract

**Background:**

Dengue hemorrhagic fever (DHF) is observed in individuals that have pre-existing heterotypic dengue antibodies and is associated with increased viral load and high levels of pro-inflammatory cytokines early in infection. Interestingly, a recent study showed that dengue virus infection in the presence of antibodies resulted in poor stimulation of Toll-like receptors (TLRs), thereby facilitating virus particle production, and also suggesting that TLRs may contribute to disease pathogenesis.

**Methodology/Principal Findings:**

We evaluated the expression levels of TLR2, 3, 4 and 9 and the co-stimulatory molecules CD80 and CD86 by flow cytometry. This was evaluated in monocytes, in myeloid and plasmacytoid dendritic cells (mDCs and pDCs) from 30 dengue patients with different clinical outcomes and in 20 healthy controls. Increased expression of TLR3 and TLR9 in DCs of patients with dengue fever (DF) early in infection was detected. In DCs from patients with severe manifestations, poor stimulation of TLR3 and TLR9 was observed. In addition, we found a lower expression of TLR2 in patients with DF compared to DHF. Expression levels of TLR4 were not affected. Furthermore, the expression of CD80 and CD86 was altered in mDCs and CD86 in pDCs of severe dengue cases. We show that interferon alpha production decreased in the presence of dengue virus after stimulation of PBMCs with the TLR9 agonist (CpG A). This suggests that the virus can affect the interferon response through this signaling pathway.

**Conclusions/Significance:**

These results show that during dengue disease progression, the expression profile of TLRs changes depending on the severity of the disease. Changes in TLRs expression could play a central role in DC activation, thereby influencing the innate immune response.

## Author Summary

Dengue virus (DENV) infections cause a broad spectrum of clinical manifestations, ranging from self-limited fever to severe disease, such as dengue hemorrhagic fever (DHF) that can be fatal. The pathogenesis of severe dengue is associated with an inadequate immune response characterized by the over-production of cytokines and other inflammatory components. However, little is known about the role of the innate immune response in the progression to hemorrhagic manifestations. TLRs are among the most important components of innate immunity and are responsible for initiating a response against a variety of pathogens, including viruses. Recent studies suggest that TLRs may contribute to disease pathogenesis. Here we aimed to explore the role of these receptors in dengue disease progression. To this end, we examined the expression of several TLRs and of co-stimulatory molecules in monocytes and DCs from dengue patients. A link between TLRs expression and the severity of dengue was observed: patients with dengue fever express higher levels of TLR3 and TLR9 than patients with DHF. This could be crucial for the host defense against dengue virus or disease progression. In addition, expression of CD80 and CD86 was altered in DCs of severe dengue cases. We show that interferon type I production is also altered in vitro through TLR9. This suggests that dengue virus affects the interferon response through this signaling pathway.

## Introduction

Dengue is the most widespread mosquito-borne viral disease worldwide. It is estimated that 50 million dengue infections occur each year, and that 2.5 billion people are at risk of acquiring dengue virus (DENV) infection [1]. It is caused by any of the four distinct, but closely related DENV serotypes (DENV-1 to 4), that are members of the *Flaviviridae* family [2]. DENV infection may lead to a febrile illness known as dengue fever (DF) but can also result in life-threatening complications defined as dengue hemorrhagic fever (DHF) and dengue shock syndrome (DSS) [3].

Clinical and epidemiological studies have revealed that secondary infection with a heterotypic serotype is a risk factor for the development of DHF [4], [5]. Furthermore, infants born from dengue-immune mothers are at risk of acquiring severe dengue disease during a primary infection [6]. The development of severe disease has therefore been linked to the presence of pre-existing antibodies. Although the mechanisms involved in immune enhanced disease are still poorly understood, multiple *in vitro* studies have shown that antibodies promote viral entry and suppress antiviral responses, allowing a higher production of virus particles per infected cell [7]–[9]. The high infected cell mass and viral load seen early in infection together with an aberrant T cell response is believed to induce a cytokine storm which causes the hemorrhagic manifestations.

The first line of defense towards pathogens is mediated by the innate immune system. Key players in the initiation of the innate immune response are Toll-like receptors (TLRs). TLRs recognize invaders through the detection of pathogen-associated molecular patterns. To date, 10 TLRs have been described in humans, 6 of which (TLR2, 3, 4, 7, 8, and 9) are implicated in recognition of viral components, namely viral nucleic acids and proteins [10]. TLRs are abundantly expressed in monocytes, macrophages and dendritic cells (DCs) [11], [12], the main target cells of DENV, and trigger antiviral defenses such as the production of interferon and pro-inflammatory cytokines. Activation of TLR3 and TLR7 inhibits DENV replication in the monocyte cell line U937 and the human cell line HEK293, respectively [13], indicating that these TLRs possess antiviral activity towards DENV. Intriguingly, however, when DENV cell entry is facilitated by antibodies, activation of TLR-negative regulators and down-regulation of TLR4 and genes associated with TLR signaling, have been observed in the monocyte cell line THP1 [14]. These results indicate that immune suppressive mechanisms are activated through this mode of viral entry. Similar results were found in peripheral blood mononuclear cells (PBMCs) from patients experiencing secondary DHF but not DF. Furthermore, and in line with the above observations, several clinical studies have indicated that alterations in pro-inflammatory cytokine production, as observed in DHF patients, can be attributed to recognition through TLRs [5]. Taken together, the recognition and subsequent activation of TLRs may be a contributing factor in dengue disease pathogenesis. In this study we examined the expression level of TLR2, 3, 4 and 9 and of the co-stimulatory molecules CD80 and CD86 in dengue patients experiencing distinct disease manifestations. TLRs and CD80/CD86 expression was evaluated in the acute and convalescent phase of the disease. The expression patterns of the different TLRs were assessed in monocytes [15], [16], plasmacytoid DCs (pDCs) [17]–[19] and myeloid DCs (mDCs) [20], [21], [16]. These cells represent important targets of DENV infection [22], and are key players in the innate immune response [23]. Our data indicate that there is a differential regulation of TLR expression profiles during the acute phase of DF and DHF.

## Materials and Methods

### Ethics statement

The protocols for patient enrollment and sample collection were approved by the Committee of Bioethics Research of the Sede de Investigación Universitaria, Universidad de Antioquia (Medellín, Colombia), as well as by the informed consent form, according to the principles expressed in the Declaration of Helsinki. All subjects read and signed an informed consent (including healthy donors). When the participant was a minor, the informed consent was signed by at least one parent.

### Study populations and blood samples

Thirty DENV-infected patients, 13 female and 17 male subjects between 12–72 years of age were enrolled in this study. Twenty healthy individuals, 10 females and 10 males 13–52 years old, were included as healthy controls (HCs). All HCs were negative for the DENV NS1 antigen and DENV IgM/IgG antibodies and had not been vaccinated against yellow fever virus. Dengue patients were enrolled from May 2009 to February 2010 in five healthcare centers located in Turbo and Apartadó, two municipalities of Antioquia, Colombia.

**Figure 1:**
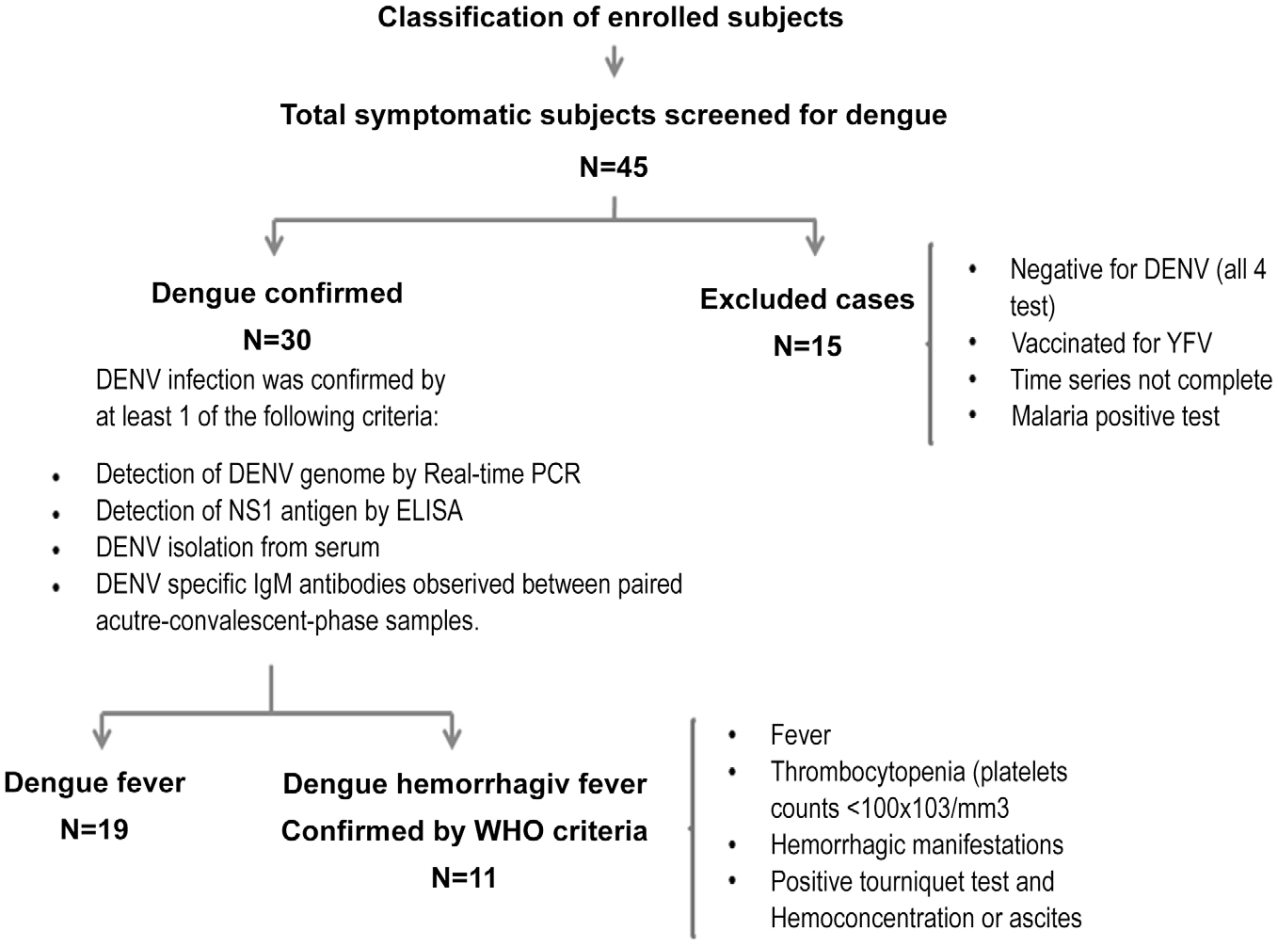
Flowchart of enrolment, inclusion/exclusion criteria, diagnosis and classification of dengue patients.

Thirty ml of peripheral blood (PB) collected in EDTA-containing tubes. Blood samples were collected three times, on the 3^rd^ and 5^th^ day after the beginning of symptoms (acute samples), and 15 days after admission to the hospital (convalescence samples). Infection with DENV was confirmed if one of the following tests was positive, (1) Platelia Dengue NS1 Antigen kit (Bio-Rad Laboratories, Marnes La Coquette, France), (2) DENV specific real-time RT-PCR [24], (3) DENV IgM detection by ELISA UMELISA (Centro de Inmunoensayo, Instituto Pedro Kourí, La Habana, Cuba) or (4) virus isolation and propagation in C6/36 mosquito cells [25]. To determine whether the patient had a primary or secondary infection, the presence of dengue-specific IgM/IgG antibodies was evaluated, using the PanBio Dengue Duo Cassette System (PanBio Ltd, Queensland, Australia). Anti-dengue IgG levels were determined on days 3, 5 and 15 after the beginning of symptoms, and if there was no rise in IgG titer over time it was considered as secondary infection. Dengue cases were classified as DF or DHF according to the 1997 guidelines of the World Health Organization (WHO) [3], we applied the old guidelines since the new WHO guidelines published in 2009 are more directly focused on clinical practice and are not widely accepted for use in research [26]. Clinical characterization of DHF included the following criteria: fever, thrombocytopenia (platelet counts <100×10^3^/mm^3^), hemorrhagic manifestations, positive tourniquet test, and hemoconcentration (20% changes in hematocrit value) or ascites as evidenced by plasma leakage. A flow chart explaining the inclusion/exclusion criteria is depicted in Figure 1.

### Flow cytometry

**Figure 2:**
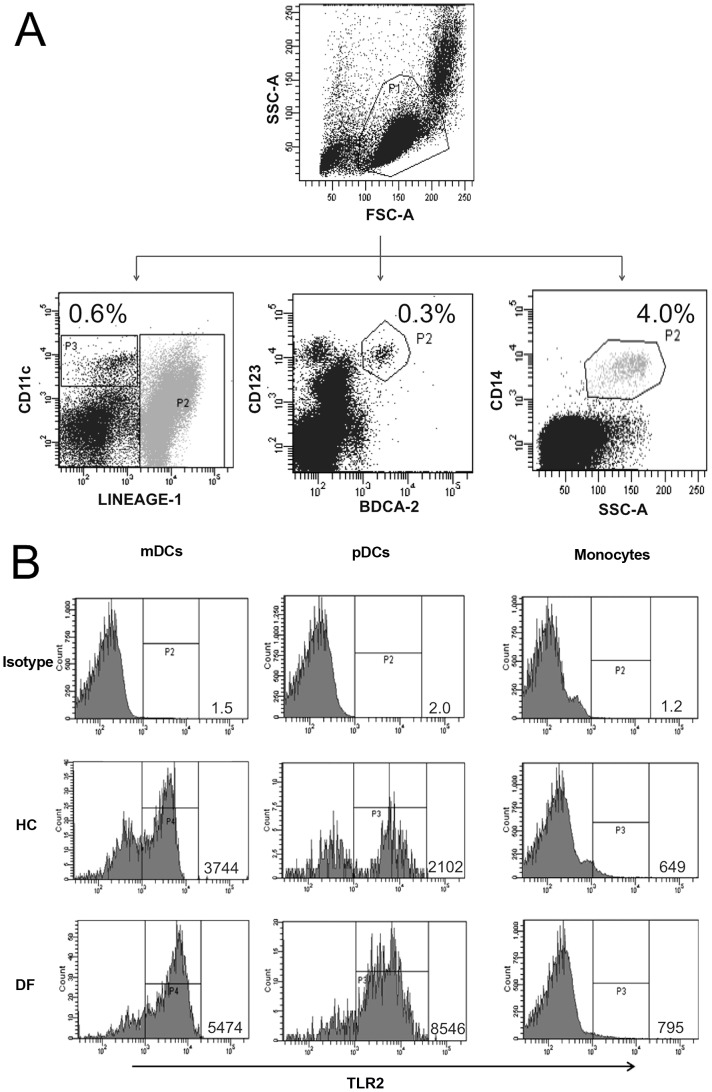
Gating strategy for identification of mDCs, pDCs and monocytes from PB samples and TLR staining. (A) Non duplets population was fractioned in mDCs as mononuclear cells Lin^−^ and CD11c ^High^ (P3); (pDCs) as CD123^+^ BDCA2^+^ (P2) and monocytes as CD14^+^ (P2) . (B) Representative examples of TLR2 expression in mDCs, pDCs, and monocytes. HC indicates healthy control and DF denotes for dengue fever.

**Figure 3:**
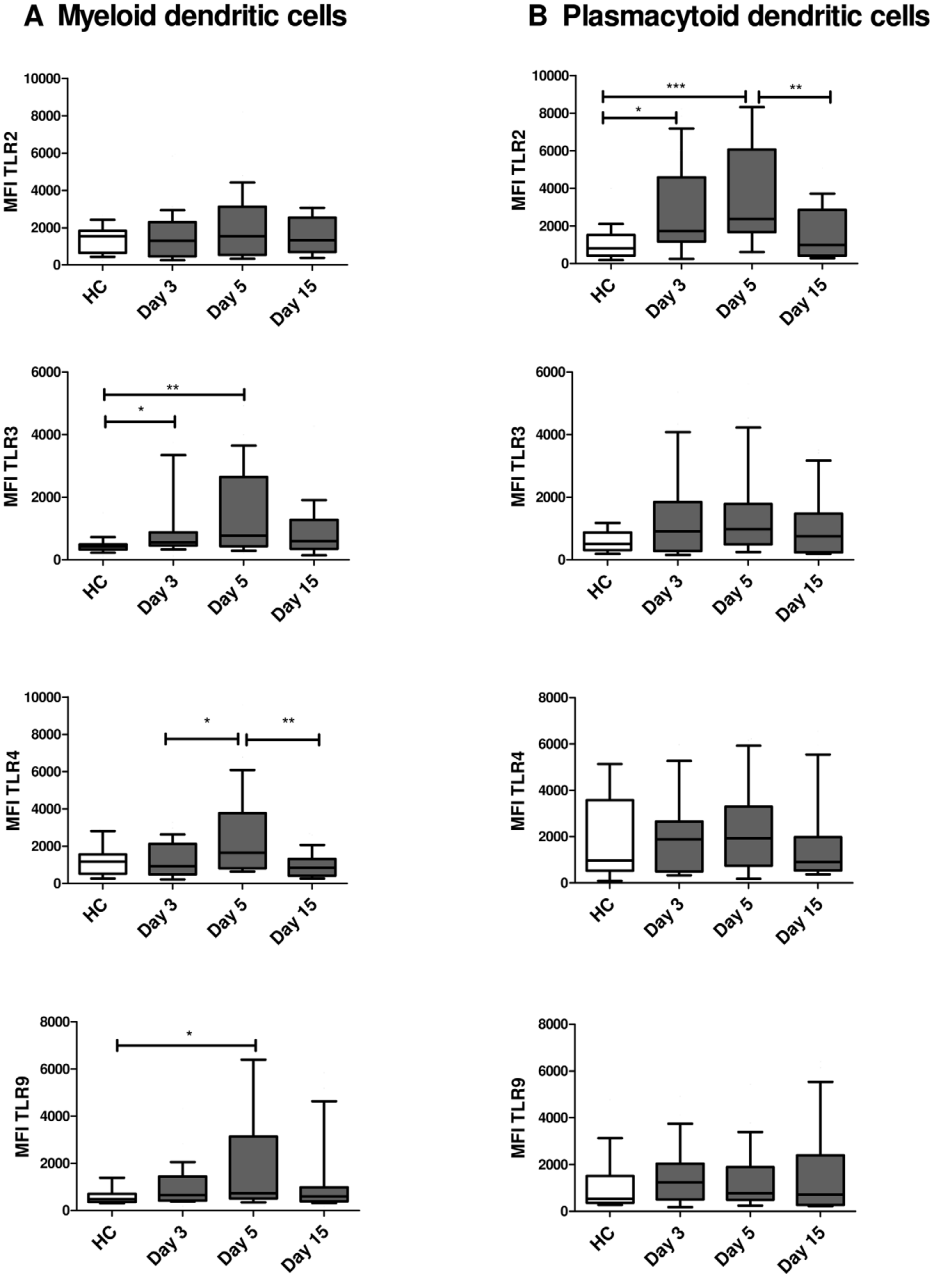
Increased expression of TLRs in mDCs and pDCs of dengue-infected patients. Expression profile of TLR2, 3, 4 and 9 in mDCs (A) and pDCs (B) of dengue patients isolated at different times after the onset of disease symptoms (day 3, 5 and 15) (*n = *30) and of healthy controls (*n* = 20). The evaluation of TLR expression was performed by flow cytometry based on mean fluorescence intensity (MFI) analysis. Data are presented as the median and 25–75 interquartile ranges Statistical analysis was performed by using the Kruskal-Wallis test followed by the Dunn's Multiple comparisons Test. *p<0.05, ** *p*<0.01 and ***p<0.001.

**Figure 4:**
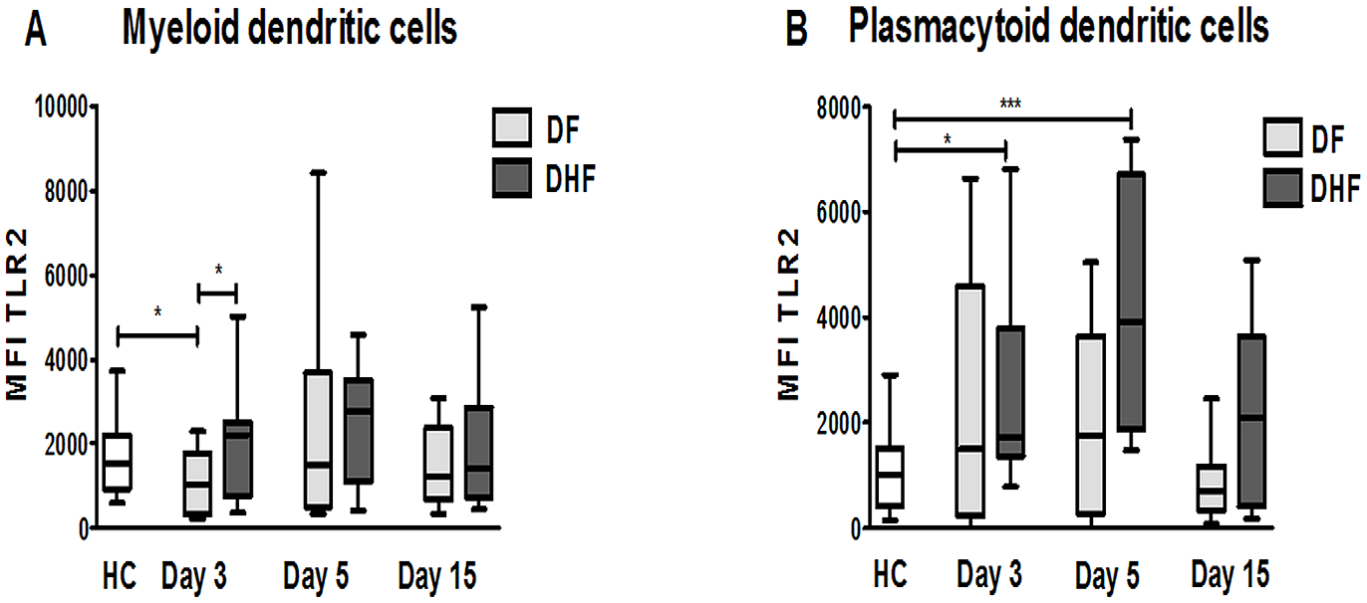
Increased TLR2 expression in pDCs of patients with severe disease. Analysis of TLR2 expression by flow cytometry of PB mDCs (A) and pDCs (B) in DF patients (n = 19) and DHF patients (n = 11) at different times after the onset of symptoms (days 3, 5 and 15). Data are presented as the median and 25–75 interquartile ranges. Statistical analysis was performed by the Mann Withney test.*p<0.05, ** p<0.01 and ***p<0.001.

PB samples were used to determine the frequency of mDCs, pDCs and monocytes and their expression of TLRs. In all patients and healthy controls the proportion of mDCs, pDCs, and monocytes among blood mononuclear cells was 0.5% (0.4–1.6%) 0.3% (0.1–1%), and 6% (4–12%), respectively. PB was incubated with the appropriate antibodies at room temperature for 25 minutes. The whole blood was lysed with lysing Buffer (BD Biosciences, San Jose, CA) during 10 minutes at room temperature. Leucocytes were resuspended and washed in PBS containing 0.5% BSA and 0.1% sodium, fixed with 2% formaldehyde and stored at 4°C until analysis. For staining of intracellular receptors (TLR3 and TLR9), the cells were treated with fixation permeabilization buffer (eBiosciences, San Diego. CA) following manufacturer's recommendations. All samples were evaluated within 2–4 h of staining and 200,000 events were acquired per tube. Analyses were performed using the BD FACS Dive V6.1.1 (BD Biosciences), and the operating software on the FACS Canto II flow cytometer. TLR levels were reported as mean fluorescent intensity (MFI) compared to the isotype control. Logical gating was used to identify mDC, pDC and monocyte populations. The acquisition gate (P1) was common for all the populations and was established based on forward scatter (FSC) and side scatter (SSC) corresponding to the gate of mononuclear cells (approximately 130,000–170,000 events). For phenotyping of each sub-population a single tube analysis was performed. The following strategy was used: mDCs (Lin1^−^/CD11c^high^) were gated as P3; Lin1 positive cells were excluded from the analysis (gate P2). The pDCs (BDCA-2^+^/CD123^high^) were gated as P2. Monocytes were identified as CD14+ versus side scatters and gated as P2 (Fig. 2A). Each specific sub-population was plotted as a histogram to show the expression of TLR2 (Fig. 2B), 3, 4 and 9. The data are presented as overall MFI for TLR on the TLR+ subset, after subtraction of isotype staining background. The following monoclonal antibodies were used: Lineage 1 FITC (anti-CD3, anti-CD14, anti-CD16, anti-CD19, anti-CD20, and anti-CD56 cocktail), anti-CD123 PE-Cy5, anti-CD11c PE-Cy5, anti-CD80 PE and anti-CD86 PE (BD Biosciences, San Jose, CA). Anti-TLR2, 3, 4 and 9 were PE conjugates (eBiosciences). Anti-BDCA-2 FITC was from Miltenyi Biotec (Auburn CA). Unstained cells and conjugated isotype antibodies were used as controls; all of them matched for concentration with the primary antibodies. All reagents were used according to manufacturer's instructions.

### Virus stocks and titration

The DENV-2 New Guinea C strain (NGC) was provided by the Center for Disease Control (CDC, Fort Collins, CO), and propagated in the C6/36 mosquito cell line. C6/36 cells were grown in L15 medium (Invitrogen, Carlsbad. CA) supplemented with 10% heat-inactivated fetal bovine serum, 1% vitamins, 1% L-glutamine and 1% non-essential amino acids (Sigma-Aldrich Chemical Co, St. Louis, MO), and incubated at 34°C without CO_2_ for 24 h. The cells were inoculated with DENV-2 at 0.01 multiplicity of infection (MOI) and incubated at 34°C for 5–6 days. The supernatants were collected and clarified by centrifugation (1500 g, 10 min). The virus titer was determined by conventional plaque assay in kidney rhesus monkey cells, (LLC-MK2), essentially as described [27]. Inactivation of the virus was achieved by exposure to UV light for 60 min.

Influenza A virus (IAV) strain A/PR8/34 was kindly donated by Paula Velilla (Immunology Group, Universidad de Antioquia). Virus titration was performed by limit dilution method, using 96-well microplates (Nunclon, NY). The virus titer was estimated by the cytopathogenicity of the cells and expressed as 50% tissue culture infectious doses/ml (TCID_50_/ml) of IAV; 1×10^4^ TCID_50_/ml were used for the infection of PBMCs. The supernatant from IAV-infected PBMCs was used as a positive control to measure interferon concentration by ELISA (eBioscience).

### Preparation and infection of PBMCs

PBMCs were isolated from HCs by density gradient centrifugation using Ficoll-Hypaque (Histopaque 1077, Sigma Aldrich Chemical Co., St. Louis, MO); all samples were processed within the first 8 h after collection. PBMCs were cultured at 1.0×10^6^ cells/ml in 24-well polystyrene tissue culture plates at 37°C in 5% CO_2_, using RPMI 1640 medium (BioWhittaker, Walkersville, MD) supplemented with 10% heat-inactivated fetal bovine serum, 100 U/ml penicillin/streptomycin (Sigma Aldrich Chemical Co.) and 1% of L-glutamine (Sigma Aldrich Chemical Co.). Subsequently, the cells were challenged with wild-type DENV or UV-inactivated (iDENV), at a MOI of 5, for 2 h at 37°C in 5% CO_2_. The PBMCs were then washed and incubation was continued for another 24 h in the presence or absence of a TLR agonist: 50 µg/ml of polyinosinic∶polycytidylic acid [poly(I∶C)], 10 µg/ml of lipopolysaccharide (LPS), 5 µg/ml resiquimod (R848) and 5 µM oligonucleotides with motifs of unmethylated cytosine-phosphate-guanine (CpG) dinucleotides type A (CpG A; Invivogen), for TLR3, TLR4, TLR7/8 and TLR9, respectively. To neutralize the stimulatory effect of CpG ODNs, 5 mM ODN TTAGGG was used (Invivogen). The supernatants were harvested after 24 h of culture and the interferon alpha (IFN-α) concentration was measured by ELISA according to the manufacturer's protocol (eBioscience).

### Statistical analyses

Statistical comparisons between the HCs and the dengue groups were performed using the Kruskal-Wallis test, with a confidence level of 95% followed by Dunn's multiple comparison test. To compare TLR expression according the severity of the disease, a Mann-Whitney test was employed. The critical value for statistical significance used for the analyses was *p*<0.05. All the analyses were carried out using the GraphPad prisma software (Graph Pad, CA).

## Results

### Characteristics of patients

In this study, 45 patients were enrolled, 30 of which were positive for DENV infection. From the 30 dengue positive cases, 19 (67%) were classified as DF- and 11 as DHF-patients (Fig. 1), based on the guidelines of the WHO [3]. The patients were admitted to the hospital and PB samples were taken on days 3, 5, and 15 after onset of symptoms. Six patients (26.6%) developed ascites related to dengue infection; there were neither patients with DSS nor mortal cases. The demographic and clinical information of the 30 dengue patients enrolled in this study are summarized in Table 1.

**Table 1:** Demographic and clinical characteristics of 30 individuals with diagnosis of dengue and 20 healthy controls.

	Controls	DF*	DHF*
	n = 20	n = 19	n = 11
Age, yr, mean ±SD	32.61(16.41)	30.94(18.24)	33.09(12.56)
***Gender***			
Male	10	11	6
Female	10	8	5
***Severity criteria, no. (%)***			
Thrombocytopenia*		3(15.78)	11(100)
Hemoconcentration		8(42.10)	9(81.81)
Ascites		2(10.52)	4(36.36)
Spontaneous hemorrhage		4(21.05)	8(72.72)
Positive tourniquet result		5(26.31)	9(81.81)

Units specified in parenthesis are percentages; otherwise data are numbers. (DF)* = Dengue fever; (DHF)* = Dengue Hemorrhagic fever; Thrombocytopenia* = Platelet counts <100000/mm3; Hemoconcentration* = Hematocrit level rising ≥20%; Ascites* = accumulation of fluid in the peritoneal cavity confirmed by abdominal ultrasound; Spontaneous hemorrhage = nose bleeding, gastrointestinal bleeding, ocular bleeding, and/or bleeding gums; Positive tourniquet = petechiae of ≥20 spots in a 2.5-cm^2^ area on the forearm after application of pressure at the midpoint between systolic and diastolic pressure for 5 min using a sphygmomanometer.

### TLR expression is up-regulated in mDCs and pDCs during the acute phase of dengue

DCs are not only important target cells of DENV infection; they also play a central role in the innate antiviral response, through TLR activation. Therefore, we evaluated the expression levels of TLR2, TLR3, TLR4 and TLR9 in mDCs, pDCs and monocytes of dengue patients, and compared them to the HCs. In mDCs of dengue patients, TLR3, TLR4 and TLR9 reached maximum expression on day 5 of illness (Fig. 3A). The MFI results showed that expression of TLR3 in mDCs was significantly higher in dengue patients than in HCs, on days 3 and 5 of illness (*p*<0.05 and *p*<0.01, respectively; Fig. 3A). For TLR4, only at day 5 a significantly higher expression was observed (Fig. 3A). Expression of TLR9 in mDCs of dengue patients was significantly increased (*p*<0.05) on day 5 of illness compared to those of HCs, while expression of TLR2 in mDCs did not differ between dengue patients and HCs (Fig. 3A). Our results also show that on day 15, the level of expression of all the TLRs tested decreased to levels similar to those of HCs (Fig. 3A).

Unlike in mDCs, in pDCs of dengue patients, only TLR2 expression was stimulated. The MFI of TLR2 reached maximum peaks on day 5 of illness. (Fig. 3B). On day 15, TLR2 expression was similar to that of HCs (Fig. 3B), suggesting that after DENV infection, TLR2 expression tends to normalize. In pDCs, the expression of TLR3, 4 and, 9 did not differ between dengue patients and HCs (Fig. 3B). In monocytes, no differences in TLR expression levels were detected between dengue patients and HCs (data not shown). Similar results were obtained when the TLR frequency was evaluated on the basis of absolute cell counts (data not shown).

### Differential expression of TLR2 in mDCs and pDCs of DF and DHF patients during the acute phase of infection

The development of DHF is associated with an increased level of circulating pro-inflammatory cytokines and chemokines [28]–[30], and several *in vitro* studies indicate that TLRs may contribute to this phenomenon [5], [31]. Therefore, we compared the expression profiles of TLR2 and TLR4 in mDCs, pDCs and monocytes of patients with DF and DHF on different days of disease to those of HCs. Similar TLR2 and TLR4 expression levels were detected in monocytes of DF, DHF patients and HCs (data not shown). In contrast, there was a modulation in TLR2 expression in DCs of DF and DHF patients. On day 3 of illness, DF patients presented significantly lower TLR2 expression in mDCs, compared to HCs and DHF patients (*p*<0.05; Fig. 4A). On days 5 and 15, the level of TLR2 expression was similar in patients with DF and DHF. In pDCs of DHF patients, a significant increase in TLR2 expression was seen on day 3 of illness, compared to HCs (*p*<0.05; Fig. 4B). This increase reached a maximum on day 5 of acute infection (*p*<0.001). No differences were found in TLR4 expression levels in mDCs (data not shown).

### TLR3 and TLR9 expression is higher in DCs of DF than in those of DHF patients

**Figure 5:**
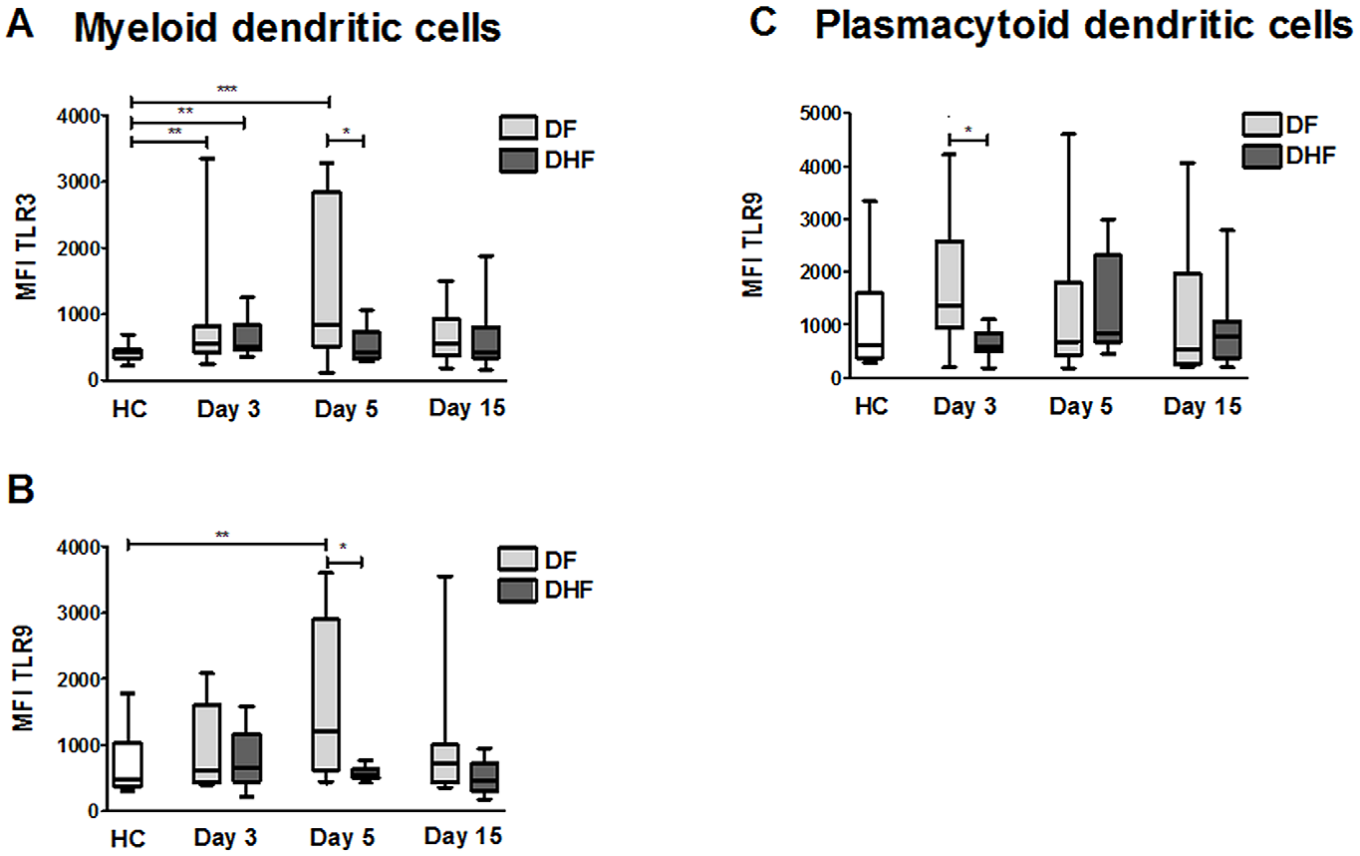
TLR3 and TLR9 in mDCs and TLR9 in pDCs have different expression levels depending on disease severity. Expression profile of TLR2 and TLR9 in mDCs (A and B) and pDCs (C) of dengue patients isolated at different times after the onset of disease symptoms (day 3, 5 and 15) (*n = *30) and of healthy controls (*n* = 20). The evaluation of TLR expression was performed by flow cytometry based on mean fluorescence intensity (MFI) analysis. Data are presented as the median and 25–75 interquartile ranges Statistical analysis was performed by using the Kruskal-Wallis test followed by the Dunn's Multiple comparisons Test. *p<0.05, ** p<0.01 and ***p<0.001.

The effect of DENV on intracellular TLR expression was evaluated in cell lines and in animal models [32], [33]; however, the effect of DENV infection on the modulation of intracellular TLR expression in DCs of patients with DF or DHF has not been reported. The results show that on day 5 after the beginning of symptoms, mDCs of DF patients had a higher MFI for TLR3 and TLR9 than mDCs of DHF patients (*p*<0.05) and HCs (*p*<0.001 and *p*<0.01), respectively; Fig. 5A and B). On day 3 of illness, mDCs from DF or DHF showed a higher MFI for TLR3 and TLR9 compared to those of HCs (Fig. 5A). In the convalescent phase, the expression levels of TLR3 and TLR9 decreased to similar levels as in HCs. The MFI results for TLR9 in pDCs of DF patients showed higher expression levels on day 3 of illness compared to those of DHF patients (*p*<0.05; Fig. 5A). Taken together, our results are consistent with recently published *in vitro* data [13], [32], [33] and suggest that the increase in TLR3 and TLR9 expression in DF patients could act as an antiviral factor.

### Down-modulation of CD80/CD86 in mDCs of DHF patients

**Figure 6:**
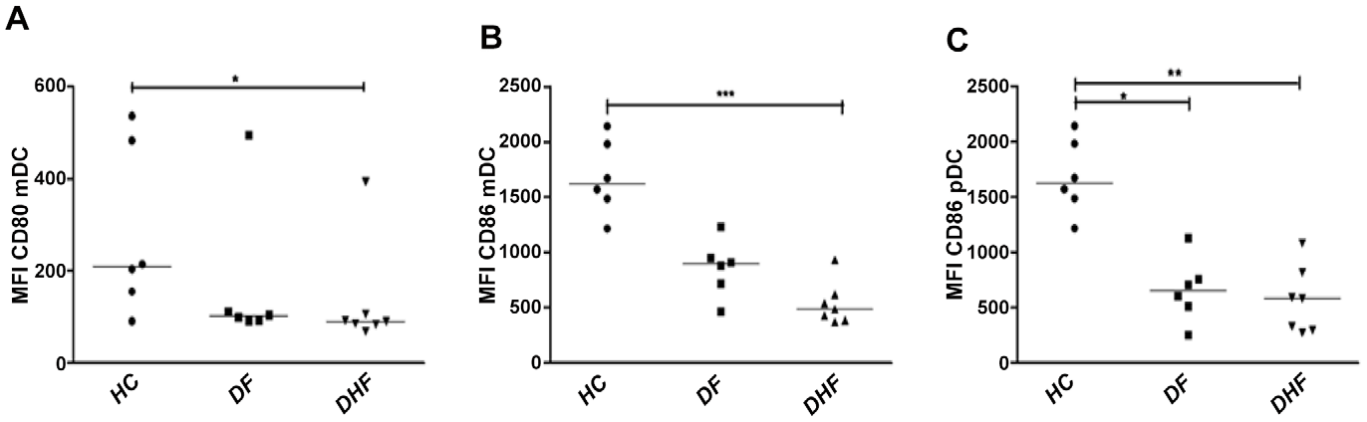
Expression of the co-stimulatory molecules CD80 and CD86 is affected in mDCs of DHF patients. Expression of CD80 (A) and CD86 (B) was evaluated by flow cytometry based on the FMI analysis in mDCs and pDCs (C) of DF and DHF patients on day 3 of illness and compared to that of healthy controls (HC). Data are presented as the median and the statistical analysis of the expression of CD80 and CD86 was performed by the Mann Withney test.*p<0.05, ** p<0.01 and ***p<0.001.

Infection of mDCs with DENV has been reported to induce DC maturation and activation, albeit to lower levels than observed in uninfected DCs [34], [35]. Since the effect of DENV on DC maturation is unclear *in vivo*, we assessed here the maturation state of the mDCs and pDCs by examining the expression profile of the co-stimulatory molecules CD80/CD86 in DF and DHF patients. Because the co-stimulatory molecule CD80 appears to be expressed in resting unstimulated mDCs [36], we quantified the expression level of CD80 in mDCs of patients with DF and DHF and compared it with the expression in mDCs of HCs. Analysis of the MFI showed that the mDCs of DHF patients express significantly lower levels of CD80 (*p*<0.05) and CD86 (*p*<0.001), when compared to HCs (Fig. 6A and 6B). Likewise, in pDCs, a significant decrease in the expression level of CD86 was seen in patients with DF and DHF (*p*<0.05 and *p*<0.01, respectively), compared to HCs. No differences were found in the expression of CD80 in pDCs (data not shown). Taken together, we observed low expression of TLR3, TLR9, CD80/CD86 in DCs of DHF patients.

### Type I IFN production through TLR9 is impaired in DENV infection of PBMCs in vitro

**Figure 7:**
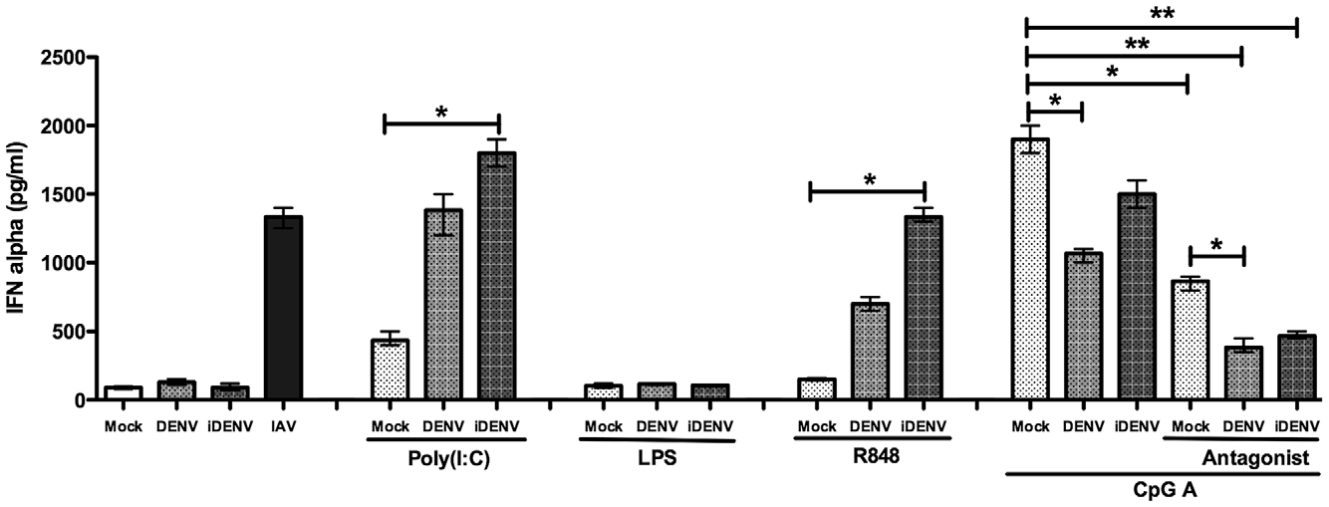
IFN-α production by PBMCs in response to TLR9 ligand is impaired by DENV infection. PBMCs from healthy controls were challenge with wild type DENV or iDENV at a MOI of 5 and then stimulated with agonists for TLR3 (poly∶IC), TLR4 (LPS), TLR7 and TLR8 (R848) and TLR9 (CpG A). The production of IFN-α was measured by ELISA a 24 h post-stimulation. The antagonist of TLR9 (TTAGGG ODN) was used to neutralize the stimulatory effect of GpG A. The supernatant from C6/36 cells were used as mock and Influenza virus was used as a positive control for IFN-α production.. Data are presented as the mean of values of at least two independent experiments performed in triplicate; error bars indicate standard deviation. Statistical comparisons among groups were carried out using the Kruskal-Wallis test comparing between groups. *p<0.05 and** *p*<0.01.

It is known that DENV is a weak inducer of type I IFN production after infection of human DCs. Several possible mechanisms have already been proposed to explain this observation [37]–[39]. The differential expression profiles of TLR3 and TLR9 in DF and DHF patients presented here may also influence type I IFN secretion. However, whether recognition of viral components by TLRs triggers IFN production in DENV-infected cells, or, whether DENV infection affects the function of TLRs is largely unclear. To expand our knowledge on this subject, we tested the ability of PBMCs to respond to the TLR3, TLR4, TLR7/8 and TLR9 ligands, and to trigger the IFN-α pathway. To this end, cells were infected with wild-type DENV or inactivated DENV (iDENV) and 2 h post-addition of the virus, the specific TLR ligands were added to the cells. We decided to uses PBMCs for these studies to mimic natural infection thereby allowing cross talking between cell subsets. PBMCs treated with the TLR3, TLR7/8 and TLR9 agonists, and challenged with DENV, showed variable but enhanced IFN-α production when compared to of non-treated cells, except for the TLR4 agonist LPS, which produced similar levels of IFN-α as the control (Fig. 7). PBMCs infected with DENV in the presence of the TLR3, and 7/8 agonists further stimulated IFN-α production (p<0.05). The observation that iDENV produces a more robust IFN-α response than wild-type DENV is in line with prior published data indicating that the nonstructural proteins, NS2B, NS4B and NS5 are responsible for inhibition of type I IFN production [37]–[39]. Interestingly, DENV infection of PBMCs in the presence of the TLR9 agonist CpG led to a significant decrease of IFN-α expression (*p*<0.05), compared to PBMCs treated with CpG A only. To investigate the potential effect of TLR9 in more detail, we next infected PBMCs in the presence of CpG A and the TLR9 antagonist TTAGGG ODN, which neutralize the stimulatory effect of CpG A. The ligands were added 2 hours post-infection with DENV. The addition of antagonist indeed decreased IFN-α production when compared to cells treated with agonist only (p<0.05). Interestingly, a stronger reduction in IFN-α production was observed in the presence of the virus (Fig. 7; p<0.01) which may suggest a combined effect between DENV and the antagonist, in the IFN-α reduction. However, additional experiments are required to confirm this hypothesis.

## Discussion

An immune response with loss in the homeostasis of cytokine secretion has been proposed as the central event in the development of DHF [40]. TLRs are important initiators of cytokine production and in this report we show that DENV can modulate/alter TLRs expression in DCs. We observed increased expression level of TLR3 and TLR9 in mDCs, and of TLR2 in pDCs, during the acute phase (days 3–5 after onset of symptoms) of DENV infection. When dengue patients were classified according the clinical outcome, a higher expression level of TLR2 was seen in DHF patients when compared to DF patients. In contrast, mDCs of DF patients expressed higher levels of TLR3 and TLR9 than those of DHF patients, especially on day 5 of illness. In pDCs, this difference was also observed for TLR9 on day 3 of illness.

The mDCs and pDCs are both important players of the innate immune system, but vary with respect to their origin, phenotype and functional features [41], [42]. Previous studies have also indicated that these cells differ in permissiveness to DENV infection. Whereas mDCs are readily infected with DENV, pDCs are essentially non-permissive to infection [43]. Interestingly, we observed changes in TLR expression in both mDCs and pDCs during the early acute phase of illness. TLR3 expression was increased in mDCs, and this is likely related to infection of these cells since TLR3 is known to recognize dsRNAs [44]. Cell surface-expressed TLR2 was up-regulated in pDCs, presumably due to sensing of the virus at the cell surface. Indeed, even though pDCs do not support a full replicative cycle of DENV, these cells do respond to infection since phenotypic and functional changes have been described upon addition of DENV to these cells [41]. This phenomenon has also been previously reported for HIV-1, where *in vitro* studies showed up-regulation of TLR2 and TLR4 in pDCs [17], [18].

Notably, we also found an up-regulation of TLR9 in mDCs. Basal expression of TLR9 has not been previously reported in mDCs, but a recent study showed that mDCs respond to the TLR9 agonists CpG A and CpG B thereby increasing the expression levels of CD80 and of human leukocyte antigen (HLA-DR9) molecules on the cell surface [16]. These findings suggest that the expression of TLR9 can be enhanced after stimulation with its agonist. We show here that IFN-α production was significantly affected in PBMCs exposed to DENV in the presence of the TLR9-agonist, which may suggest that TLR9 responds to DENV infection. However, how TLR9 is activated in DENV infection is unclear, as TLR9 is known to recognize pathogen-associated DNA [19], [45], [46]. One could speculate that the increased TLR9 expression is due to cross-talk between different receptors or adaptor proteins from different signal pathways as has been described to occur in a pro-inflammatory environment [47].

Monocytes have also been reported to represent target cells of DENV replication [48]. Moreover, Azeredo *et al.* [31] showed an increase of TLR2 expression in monocyte CD14+ of dengue-infected patients. Subsequent *in vitro* studies revealed an increased frequency of TLR2 in pro-inflammatory monocytes (CD14+ and CD16+) and it was proposed that overexpression of TLR2 in CD16+ cells could contribute to DHF. We also assessed TLR2/TLR4 expression in monocytes but no effect of DENV infection on TLR2 and TLR4 expression was detected. This discrepancy is probably related to differences in the analysis and classification of the cell populations.

TLR3 is specifically up-regulated in mDCs in patients with acute DF since no TLR3 up-regulation is seen in patients that develop DHF. Previously, Tsai and co-workers [13] reported that TLR3 induces an anti-dengue response in HEK293 cells. Furthermore, there is evidence that the type I IFN response initiated by TLR3 contributes to the elimination of Hepatitis C virus [49]. Based on the above observations, we postulate that TLR3 is associated with antiviral responses against DENV, promoting the production of pro-inflammatory cytokines and type I IFN. This inhibition of DENV could prevent the development of severe manifestations. Indeed, and in line with our results, a recent report showed down-regulation of TLR3, TLR4 and TLR7/8 in PMBCs of patients experiencing secondary DHF but not in patients expressing DF [14].

TLR2 expression was up-regulated in pDCs and mDCs of DHF patients but not of DF patients. Recent studies suggest that TLR2 is an important promoter of pro-inflammatory cytokine release, (reviewed in [46], [47]). Increased pro-inflammatory cytokine production is one of the hallmarks of severe disease [50], [51]. Therefore we postulate that individuals with a higher response to DENV through TLR2 may be more likely to develop severe manifestations, whereas patients who express TLR3 and TLR9, may have some degree of protection and may probably be less likely to develop DHF.

TLRs not only promote cytokine release; they also promote the up-regulation of co-stimulatory molecules such as CD80 and CD86 to favor cell maturation and efficient antigen presentation to T cells [52]. We found that mDCs of DHF patients have lower expression levels of CD80 and CD86 than HCs suggesting inefficient maturation of mDCs in these patients. This may be explained by the low TLR3 and TLR9 expression level in patients with DF or DHF. In pDCs, a significant down-regulation of CD86 was observed in both DF and DHF patients. This could have important consequences on the development of a specific immune response able to induce memory T cells to control future infection. This hypothesis is supported by the observation of lower CD80/CD86 expression levels in DHF patients, compared to DF patients (in mDCs) and HCs (in DCs). Libraty *et al.* [34] reported lower expression of CD80 and CD86 in DCs in DENV infection *in vitro*. Sun *et al*. (2009) observed that in purified pDCs and mDCs the presence of the virus promotes the expression of CD80 and CD86 in mDCs but not in pDCs suggesting that DENV can down regulate the co-stimulatory molecules CD80 and CD86 [43]. However, further studies are required to elucidate the mechanisms involved in this phenomenon.

In conclusion, the differential expression of TLRs in dengue may influence the clinical outcome of the disease. Future research is necessary to fully understand the participation of the innate immune response in dengue pathogenesis and to assess the possibility of using TLR agonists as vaccine adjuvants in dengue vaccines.
